# Doing comic geographies

**DOI:** 10.1177/1474474016630967

**Published:** 2016-02-14

**Authors:** Phil Emmerson

**Affiliations:** University of Birmingham, UK

**Keywords:** comedy, humour, incongruity, reflexivity, qualitative methods

## Abstract

This article reflects on how notions of ‘the comic’ may be of added value to geographers’ research. It is formed around the idea that there are aspects of space and society that are by nature incongruous and unsuitable to be understood through frameworks of scholarship that privilege ‘reason’ and objectivity above all else. The author thus reflects on how these notions of ‘the comic’ as a mode of thought can be applied to understanding different fields of research. Ultimately, the article draws out how using this comic mode also forms an ‘inward’ reflective process which can help to understand the often complicated positions that researchers hold. This article thus calls for an inclusion of the often otherwise ignored comic aspects of the world into scholarship so that we, as geographers, may provide fuller and more human critical analyses of space, culture and society.

## Introduction

Over a decade ago, Mike Crang noted that the ‘comic’ is not a sensibility that academic prose is very good at evoking,^1^ although he offered few insights into how or why it might be useful to geographers. This article explores my own methodological engagements with comic modes of thought, particularly as a practice of reflexivity – an idea which catalysed around participation in a ‘learn stand-up comedy’ course as part of a geographical research project into the production of stand-up comedy. It reflects on what the practices of producing comedy may contribute to geographers’ understandings of the relationships between ourselves and our research. The article is structured in three parts: I briefly offer my story, before highlighting instances where the comic has provided a means of critical insight. Finally, I reflect on how the comic has helped me to understand my positionality, both within my research and more generally. Connecting the final two sections is the concept of incongruity, a key idea within the production of comedy which I in turn argue can also serve a purpose within critical geographical methodologies.

## Comedy and me

Comedy is a highly complex socio-cultural phenomenon, and while a full discussion of its types, theories and applications is beyond the scope of this article, it is worth noting that there are a number of academics who have provided analyses of this type, with notable contributions coming from Critchey,^2^ Billig^3^ and Double,^4^ as well as a small number who have engaged with it geographically.^5^

My interest in comedy emerged through a research project that examined the use of space in the production of comedy, looking to understand the differences and similarities with other performance art forms. My initial intention was not to ‘do’ stand-up myself, but through the project a perceived ‘need’ evolved for me to experience performing first hand. I enrolled on a ‘learn stand-up comedy course’ run by a local professional comedian which involved 2-hour sessions, every Monday, for 12 weeks. The first 6 weeks covered comedy writing techniques, including exaggeration, anthropomorphisation and punning; the second six, performance: how to direct jokes, using the body and emotions; remembering sets; and dealing with ‘hecklers’. The final 30 minutes of each session provided a chance to perform and get feedback on our acts.

In total, I performed six times, before finding it too emotionally draining and stopping. My initial performance was part of ‘Bright Club’ – a national series of events where academics perform stand-up comedy about their work – and the other five were at ‘open mic’ events where I performed the comedy-geography set that I developed for Bright Club – although with less success.

Bright Club in many ways epitomises a rationale for engaging with comedy academically. It operates on the principle that comedy can bridge the gap between ‘the academy’ and ‘the public’, showing some success in this regard. My experience of comedy, however, has led me to examine the actual construction of the comic, recognising a parallel between this process and that of critical thought.

## Comical critique

In *The Order of Things*, Foucault describes how his *laughter* on reading Borges ‘broke up the surfaces and planes to which people were accustomed, disturbing and threatening the distinction between Same and Other’.^6^ The humour that Foucault sees is part of an active critique, taken up by Butler, who argues in *Gender Trouble* that through comical *parody*, politics can be critiqued and recast.^7^ Similarly, Haraway’s *Cyborg Manifesto* calls for the inclusion of *irony* into socialist-feminism.^8^ For Haraway, this irony is a crucial way of uncovering ‘the truth’ behind the political myth that links feminism, socialism and materialism and about finding a way to negotiate the contradictions within the popular representations of these theoretical ideologies.

Rita Barnard notes,

[P]arody serves to underscore a crucial methodological point, namely that defamiliarization – an ability to ‘look awry’, in Slavoj Zizek’s phrase – is an indispensable part of any progressive critique. More: it reminds us that a productive estrangement may result not only from erudite academic innovations and inventive interdisciplinary perspectives, but also from a humorous inversion of received ideas and pieties. The postcolonial critic, in other words, may at times be a laughing one.^9^

These arguments rest around the idea that to (metaphorically or physically) laugh is to know the world in a different way.^10^ In looking awry, Zizek asks that we explore ideas outside of ‘normal’ academic frameworks, matching them up in ways that may appear contradictory. Interestingly, this ability to look awry is also the core of humour, which despite its many different theories^11^ is at its most basic constructed around *incongruities*, where two apparently opposing ideas (such as Same and Other) suddenly collide and make sense in each other’s contexts.^12^ Within comedy as it is conventionally thought, jokes about social phenomena can often also reveal the fragility of many ‘common sense’ assumptions that structure society and maintain inequalities within it.^13^

To be comical is sometimes therefore to be critical, and as such, I argue that there is space for analysing (and constructing) incongruities within our work. For me the comic needs to be understood as one of a number of ways of representing the world and thus seen as part and parcel of doing geographical research that engages with the multiplicity of ways that different people ‘know’ and engage the often contradictory spaces in which they live. While this approach can be applied to the spaces and people that we research, jokes are often very personal things, and as such, in practice, I have found myself directing this comic mode of thought inwards, as a means of being critically reflexive.

## Turning the comical inward

The key lesson learnt from the comedy course was that your comic persona should inform everything you do. Comedians often speak about their comic persona as being ‘me, but with the volume turned up’. You take your personality, thoughts and feelings and exaggerate them, making them grotesque and unreasonable which (ideally) makes them laughable. Being unreasonable is a difficult position to hold as an ethnographic researcher however, where despite much critique, particularly from Feminist geographers, there is often still an expectation that the researcher will somehow remain detached, ‘objective’ and rational.^14^ Alas, there are implications for trying to do social science research when this is completely thrown out by the grotesque body of the researcher turned comic – a body which can be overly ignorant, stupid or just monstrous. I became very aware of this contradiction during the comedy course. I wished to present myself as a caring and considerate researcher to the other members, something achievable before and after each lesson but much harder during the lessons themselves, where often presenting myself as ignorant or intolerant to certain things was an active tactic to be funny.

As such, in my experience of being both a geographer and a comic, positionality became a particularly complex thing to negotiate. Again, the status quo of ethnographic research encourages us to be open about our position, to share it with others and reflect on it thoughtfully. While the pragmatics of this have also been challenged – for example, by researchers who actively use their femininity when interviewing elite men, to encourage more forthcoming answers to their questions^15^ – comedy exaggerates this further, often actively masking the researcher/comic’s ‘true’ position. This, however, can also be highly revealing about elements of ourselves, as I noted in my field diary while preparing for my first performance:

I never knew I had such strong feelings about the car I drive, about the way that people hand me change in shops, about politics, about the state of geography. But I’m writing jokes about them (although not good ones), all the while thinking about how I really feel. I realise these things make me sound stupid. In a way that’s the point, I am bending them, breaking them, exaggerating them. But at the base of it all, there’s some kernel of truth, there’s a part of me that I didn’t know even cared this much. (Field diary notes – 20 August 2014)

Being reflexive is something that I sometimes find difficult. There are deep hypocrisies in myself, things I’m ashamed of, things I’ve changed my mind about and things I love to dislike, and these can be hard to accept, both at a personal level and within the confines of an academic system that privileges ‘reason’ as a means of constructing knowledge. Being comical releases some of these shackles, privileging incongruities, which often seem to be disregarded by academics because they are ‘irrational’, contradictory and unable to be easily categorised. Through doing comedy, therefore, I learnt to unlock some of these aspects of myself, recognising incongruities and how my reactions to them shapes my interaction with the world. Despite often being illogical, they still formed a vital part of the world that I inhabit and thus the one I take into my research.

Being comical thus provided a new way for me to practice reflexivity. In practice, the ‘jokes’ I created through this were not very funny; to be honest, they often never actually became jokes, but it was through the process of writing and thinking about these ‘jokes’ that I could explore some of the incongruous elements of my own position both in research and my life more generally. It is this that I want to encourage geographers to do more often.

## Conclusion

My aim has been to share an idea, a sort of call for geographers to look more closely at the comical aspects of ourselves and everyday life. I’m not suggesting that we abandon serious scholarship, for it is through the serious that we can by far make the biggest impacts. Instead, I want to emphasise that the comic has value too and that representing comic elements is important, particularly in societies where the comic is integrated into almost every aspect of culture. Neither am I suggesting that everyone should do a comedy course or study comedy in depth, merely that when something makes us laugh, chuckle or just smile to ourselves: that we take note of it, think about it, play with it, distort it and see what we make of it then. It may uncover nothing, but it may reframe the way that we understand ourselves in relation to our research.

## Notes

1. M.Crang, ‘Qualitative Methods: Touchy, Feely, Look-See?’, *Progress in Human Geography*, 27(4), 2003, pp. 494–504.

2. S.Critchley, *On Humour* (London: Routledge, 2002).

3. M.Billig, *Laughter and Ridicule: Towards a Social Critique of Humour* (London: SAGE, 2005).

4. O.Double, *Stand-up! On Being a Comedian* (London: Methuen Drama, 1997).

5. See, for example, J.Ridanpää, ‘Geographical Studies of Humor’, *Geography Compass*, 8(10), 2014, pp. 701–9; H.Macpherson, ‘“I Don’t Know Why They Call It the Lake District They Might as Well Call It the Rock district!” The Workings of Humour and Laughter in Research with Members of Visually Impaired Walking Groups’, *Environment and Planning D: Society and Space*, 26(6), 2008, pp. 1080–95.

6. Described in J.Butler, *Gender Trouble: Feminism and the Subversion of Identity* (New York: Routledge, 2006), p. 139.

7. Butler, *Gender Trouble*.

8. D.Haraway, ‘A Cyborg Manifesto: Science, Technology, and Socialist-Feminism in the Late Twentieth Century’, in D.Haraway (ed.), *Simians, Cyborgs, and Women* (London: Free Association Books Ltd, 1991), pp. 149–82.

9. Page 278: in R.Barnard, ‘On Laughter, the Grotesque, and the South African Transition: Zakes Mda’s Ways of Dying’, *Novel: A Forum on Fiction*, 37(3), 2004, pp. 277–302.

10. See also M.Bakhtin, *Rabelais and His World* (Bloomington: Indiana University Press, 1984).

11. See Critchley, *On Humour*.

12. Critchley, *On Humour*.

13. See, for example, A.Amarasingam, ‘Laughter the Best Medicine: Muslim Comedians and Social Criticism in Post-9/11 America’, *Journal of Muslim Minority Affairs*, 30(4), 2010, pp. 463–77.

14. See N.Denzin, *Performance Ethnography: Critical Pedagogy and the Politics of Culture* (Thousand Oaks: SAGE, 2003).

15. L.McDowell, ‘Elites in the City of London: Some Methodological Considerations’, *Environment and Planning A*, 30, 1998, pp. 2133–46.

